# Scleroderma Renal Crisis in a Case of Mixed Connective Tissue Disease Treated Successfully with Angiotensin-Converting Enzyme Inhibitors

**DOI:** 10.1155/2021/8862405

**Published:** 2021-01-06

**Authors:** Jomana Madieh, Iman Khamayseh, Alaa Hrizat, Abdurrahman Hamadah, Kamel Gharaibeh

**Affiliations:** ^1^Department of Internal Medicine, Faculty of Medicine, Al-Quds University, Abu Dis, State of Palestine; ^2^Department of Internal Medicine, Faculty of Medicine, Hashemite University, Zarqa, Jordan

## Abstract

Mixed connective tissue disease (MCTD) is a rheumatic disease syndrome with overlapping features of scleroderma, systemic lupus erythematosus, and polymyositis. An extremely rare but serious complication that can occur in MCTD is scleroderma renal crisis (SRC). There have been different approaches to the treatment of SRC associated with MCTD. We present a case of MCTD with chronic features of Raynaud's phenomenon, dermatomyositis, and thrombocytopenia complicated with acute SRC which showed a great response to ACE inhibitors. Here, we advise the early and aggressive use of ACE inhibitors as soon as SRC is suspected.

## 1. Introduction

Mixed connective tissue disease (MCTD) is a rheumatic disease syndrome originally described in 1972 and applied to patients with overlapping clinical features of systemic sclerosis (scleroderma), systemic lupus erythematosus (SLE), and polymyositis along with the presence of high titers of a distinctive antibody to the U1 ribonucleoprotein (U1 RNP) [1, 2]. Clinical features of high frequency include Raynaud's phenomenon, arthralgias, swollen hands, fingers with a sausage-like appearance, esophageal dysfunction, and muscle weakness. MCTD can potentially affect various organ systems resulting in pulmonary, renal, cardiovascular, gastrointestinal, and central nervous system manifestations [3, 4]. Scleroderma renal crisis (SRC) is an extremely rare complication in MCTD [5, 6]. It typically presents as an accelerated hypertension of sudden onset and acute renal injury, with or without microangiopathic hemolytic anemia or thrombocytopenia [5, 7, 8]. Several reports have emphasized the use of angiotensin-converting enzyme inhibitors (ACEi) and their dramatic improvement on the outcomes and survival of scleroderma patients experiencing SRC [8–11]. However, only a few reports on the treatment of SRC in MCTD exist [5, 6, 12]. In this article, we report a rare case of MCTD complicated by SRC which was treated successfully with ACEi.

## 2. Case Report

This is a case of a 30-year-old female with a history of MCTD. She had been diagnosed with MCTD with a positive ANA: >3.5 U (3–5.9 U is positive, ≥6 is strongly positive) and high titers of anti-RNP antibody: 208.8 IU (anti-RNP > 26 U is positive) associated with Raynaud's phenomenon, dermatomyositis, chronic thrombocytopenia, and chronic arthralgia 4 months prior to her presentation. Since then, she was treated with azathioprine, prednisone, nifedipine, and naproxen. The patient was brought to the emergency room (ER) after she had experienced two syncopal episodes. Five days prior to admission, she developed a fever (*T*max: 102 F, 38.8 C) associated with recurrent nausea and vomiting. She also reported dyspnea on exertion, palpitations, and myalgia. Despite negative urinary symptoms, she was diagnosed with a urinary tract infection (UTI) based on the urinalysis (UA) findings of pyuria, hematuria, and +2 proteinuria at that time. She had no diarrhea, abdominal pain, chest pain, or new active skin changes.

Initial physical examination in the ER showed the patient to be alert and oriented. Vital signs showed heart rate (HR) of 110 bpm and blood pressure (BP) of 160/108 mmHg. Based on the clinical picture and laboratory findings, she was thought to be dehydrated and 2 liters of 0.9% saline was given intravenously. Two hours later, she developed sudden tachypnea and dyspnea with hypoxaemia and elevated BP (systolic 220 over diastolic 115) that eventually required intubation for acute hypoxemic respiratory failure. Chest X-ray (CXR) findings were consistent with bilateral pulmonary edema. The patient was placed on nitroglycerine (NG) infusion and transferred to the medical intensive care unit for further management.

Laboratory workup on admission: WBC 8.9 × 10^9/L (4–11 × 10^9), hemoglobin 13.5 g/dl (12–15 g/dL), hematocrit 40.4% (36%–47%), platelet count 165 10^3^/uL (150–400 × 10^9), serum creatinine (sCr) 1.18 mg/dL (baseline sCR was 0.56 mg/dL), serum bicarbonate 17 mmol/L (18–22 mmol/L), plasma renin activity (PRA) 143.5 ng/mL/hour (normal is ≤ 0.6–4.3 ng/mL/hour), potassium 2.3 mmol/L (3.5–5 mmol/L), haptoglobin 11 mg/dL (50–220 mg/dL), C-reactive protein (CRP) 1.9 mg/L (<5 mg/L), ESR 40 mm/hour (normal is < age/2 mm/hour), serum lactate dehydrogenase 423 U/L (50–150 U/L), arterial pH 7.08 (7.35–7.45), arterial pO_2_ 68 mmHg (75–100 mmHg), and arterial HCO_3_ 13.3 mmol/L (18–22 mmHg). Urinalysis showed pyuria, proteinuria, and hematuria including RBC casts and dysmorphic RBCs. The patient was found to be oliguric after monitoring urine output for 24 hours (total urine output was 215 ml). Spot protein/Cr ratio was 3.2.

**Figure 1:**
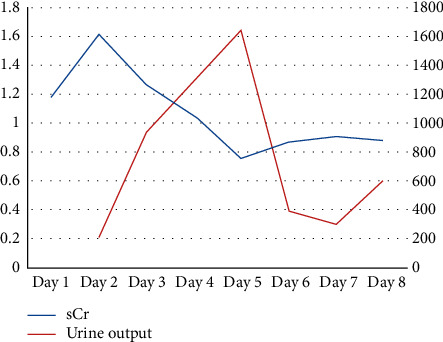
Serum creatinine trend (blue line) and urine output trend (red line) throughout patient's hospitalization.

Despite improvement in BP readings (with an IV antihypertensive medication), the patient remained oliguric and sCr continued to rise, peaking at 1.62 mg/dL, which is 3 times her baseline sCr. Given the history of MCTD along with the typical presentation of a possible SRC-like syndrome, the decision was made to initiate Captopril. Within 24 hours from starting captopril, urine output started to increase and oliguria resolved with improvement in renal function as shown in Figure 1. We titrated up the captopril and were able to wean off nicardipine.

**Figure 2:**
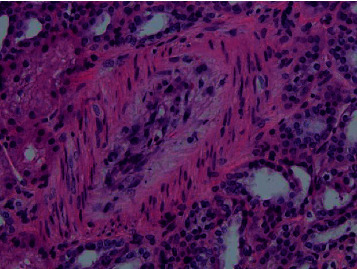
Edematous mucoid intimal thickening of interlobular artery (H&E).

**Figure 3:**
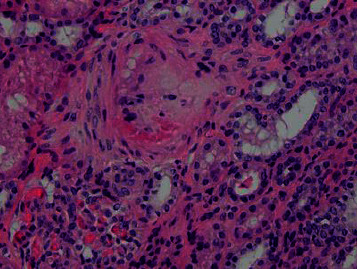
Intimal fibrinoid changes in interlobular artery (H&E).

**Figure 4:**
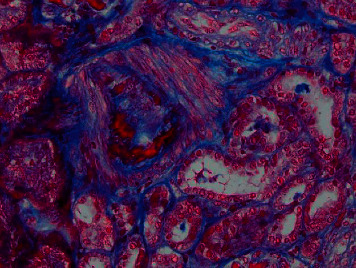
Intimal fibrinoid changes in interlobular artery (trichrome stain). Same interlobular artery is shown in figure 3.

**Figure 5:**
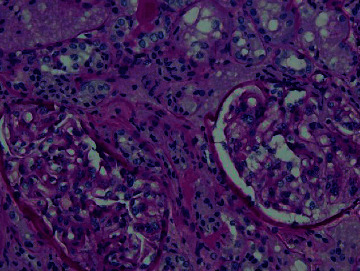
Normal glomeruli (PAS).

On the third day, once the patient was hemodynamically stable and blood pressure was under control, the decision was made to perform a renal biopsy for further workup. It showed thrombotic microangiopathic changes in the interlobular arteries which are consistent with SRC-like syndrome as seen in Figures 2–5. A day later, the patient was successfully extubated. A week later, the patient was discharged with sCr of 0.88 mg/dL and in good condition with the impression of a SRC-like syndrome in MCTD.

## 3. Discussion

The exact prevalence and incidence of MCTD remain unknown. However, it has always been reported to be more common in females despite the difference in ratios estimated by different studies [13–15].

Being an overlap syndrome, the definitive diagnosis of MCTD can be difficult to achieve [16]. The early clinical manifestations are nonspecific and the disease state is considered an undifferentiated connective tissue disease (UCTD) [16–18]. During this stage, patients commonly complain of fatigue, myalgias, arthralgias, and Raynaud's phenomenon [16]. Findings suggestive of MCTD occur sequentially evolving over the years [16, 17, 19]. The presence of Raynaud's phenomenon and high titers of anti-U1 RNP antibodies are strong predictors of future evolution to MCTD [16, 19].

As MCTD was initially described in 1972, it was thought to be a connective tissue disease syndrome that is of favourable prognosis and excellent responsiveness to corticosteroid therapy compared to other connective tissue diseases (CTD) [1]. It was suggested that antibodies to ENA (i.e., U1 RNP), which are distinct to MCTD, have a protective role [1]. At that time, renal involvement in MCTD had not yet been identified. However, since then, as more cases of MCTD were being reported, findings of cardiac, pulmonary, and renal involvement emerged and did not have as much of a favourable prognosis as initially perceived.

Multiple criteria (Sharp, Alarcon–Segovia, Khan, Kasukawa) were established for the diagnosis of MCTD based on the serological findings of high-titer-anti-U1 RNP antibodies accompanied by other clinical features of the disease. One study showed that the Alarcon–Segovia's criteria had a sensitivity and specificity of 63% and 86%, respectively [14].

Renal involvement in MCTD is uncommon (10%–26% of patients) and is often asymptomatic [5]. In a study looking at the renal involvement in MCTD, it was found that the only early indicator of renal disease was an abnormal urinalysis with no overt clinical features. Serologic studies were not a helpful predictor either. Renal involvement can occur as glomerulonephritis (GN), nephrotic syndrome, amyloidosis, and, rarely, the renal vasculopathy characteristic of scleroderma, hence the name scleroderma renal crisis (SRC) [12, 20].

SRC in scleroderma occurs in 5–10% of patients [21]. However, in MCTD, it is a severe complication that has rarely been reported [3, 5, 6, 22, 23]. It typically presents as a sudden onset of accelerated hypertension (which could often be malignant) and acute renal injury with or without microangiopathic hemolytic anemia or thrombocytopenia [1, 7, 20].

The biochemical picture of a patient with SRC includes elevated serum creatinine, microangiopathic hemolytic anemia (MAHA), thrombocytopenia, and hyperreninemia. Urinalysis commonly shows hematuria, proteinuria, and granular casts visible on microscopy [7, 8, 24, 25].

Although renal biopsies are necessary to confirm the diagnosis and exclude other concurrent pathological processes, they are not regularly requested in SRC [7]. The histological picture of SRC is thrombotic microangiopathy similar to that seen in idiopathic malignant hypertension. Primary small vessel manifestations usually predominate over glomerular changes. Histological findings may vary along the course of the disease [7] (see Table 1).

In some cases, SRC can remain asymptomatic, reflecting an ongoing subclinical renal injury [7]. The acute onset and rapid progression of renal injury could be triggered by high-dose steroids (≥15 mg/day of prednisone), diffuse skin involvement, new-onset anemia, and new cardiac events. Although the use of nonsteroidal anti-inflammatory agents has not been reported as a precipitating factor for SRC, these drugs can induce acute kidney injury (AKI). The reduced synthesis of renal vasodilating prostaglandins (PGE2 and PGI2) and, consequently, compromised renal blood flow can lead to reversible renal ischemia and AKI [25, 26]. Patients who need systemic steroids therapy should be carefully monitored for the development of SRC [8].

In this case, our patient had initially received aggressive fluid resuscitation causing a sudden elevation in her blood pressure. This may have contributed to further deterioration in her kidney function by aggravating more endothelial injury. She had also been managed for MCTD by chronic steroids (prednisone 10 mg/day) and was started on NSAIDS 4 weeks prior to admission. According to Steen [25], cautious use of NSAIDs is prudent in systemic sclerosis patients at high risk for SRC. Therefore, NSAIDs may have also contributed to the precipitation of SRC in our patient.

The treatment of SRC is based on the aggressive control of hypertension with ACEi [9, 10]. The best outcome without reaching dialysis is exhibited when ACEi therapy is given promptly and aggressively. Serum creatinine less than 265 *µ*mol/L (3 mg/dL) at the time of initiation of ACEi is also associated with favourable prognoses [10]. Consequently, ACEi therapy should be started as soon as scleroderma renal crisis is diagnosed [7, 8, 10]. MCTD patients that are experiencing features of scleroderma should be continuously screened for SRC by the regular monitoring of blood pressure and renal functions [8, 10].

To our knowledge, only nine cases of MCTD with SRC have been reported as summarized in Table 1. All patients but one [27] had features of scleroderma, most commonly Raynaud's phenomenon. Of the nine cases gathered, eight cases were treated with ACEi, three of which developed end-stage renal disease requiring chronic hemodialysis [12, 23, 27]. The patient who was not started on ACEi eventually became haemodialysis-dependent [22]. Of the five patients who responded to ACEi, three had sCr levels less than 3 mg/dl at the time of initiation of treatment [6, 23, 28]. This comes in agreement with a previous study conducted by Steen and Medsger that exhibited the best outcomes of ACEi treatment in patients with sCr concentrations less than 3 mg/dl [10].

Several causes might have led to the failure of renal recovery in the patients who received ACEi treatment [12, 23, 27]. Khalil et al. [12] suggest that this outcome in their case was attributed to preexisting chronic kidney disease, previous exposure to high-dose steroids, and delayed initiation of ACEi. Similarly, Khan et al. [23] reported a patient with a two-year history of illness that has been dealt with inadequately resulting in a late diagnosis, the development of advanced chronic kidney disease, and failure of early initiation of therapy with ACEi. Greenberg and Amato [27] reported a case of MCTD with SRC precipitated by high-dose steroids (prednisone 60 mg/day) which did not respond to ACEi treatment. In their report, they explained that corticosteroids inhibit prostacyclin production and the subsequent rise in ACE levels in patients with an underlying microangiopathy involving the kidneys is enough to cause renal failure.

## 4. Conclusion

Despite the rarity of SRC in MCTD, it should not be overlooked. A sudden rise in blood pressure or the combination of high blood pressure and acute kidney injury (with or without MAHA) in a MCTD patient should be considered SRC-like syndrome until proven otherwise. SRC-like syndrome is a serious complication which, if not treated promptly, might lead to permanent renal damage.

Several reports have emphasized the use of ACEi and its dramatic improvement on the outcomes and survival of scleroderma patients experiencing SRC. However, only a few reports on the treatment of SRC in MCTD exist. Among these reports, including our case, ACEi have shown a major role in the treatment of such crises and the prevention of permanent renal damage. We should consider ACEi a first-line treatment for SRC-like syndrome in MCTD as already documented to be a first-line treatment for SRC in patients with scleroderma. Therefore, in a patient diagnosed with MCTD, we recommend early initiation of treatment with ACEi as soon as SRC is suspected. Future retrospective and prospective studies should be done to further confirm our conclusion.

## Figures and Tables

**Table 1:** Summary of case reports of scleroderma renal crisis (SRC) in mixed connective tissue disease (MCTD).

Case report	Sex/age	Clinical background	Pathological features	Treatment	Outcome
Our case	Female/30	History of MCTD presented with vomiting received aggressive fluid resuscitation for suspected dehydration resulting in hypertensive emergency, pulmonary edema, and AKI	Biopsy revealed TMA type changes within 3 interlobular arteries. The changes compatible with malignant HTN or SRC. 1^st^ interlobular artery shows moderate edematous mucoid intimal thickening, the 2^nd^ shows intimal fibrinoid change, and the 3^rd^ shows moderate to marked intimal thickening with intimal fibrosis and mild edema	Captopril	Responded to treatment
Cheta et al. [5]	Female/54	Patient presented with shortness of breath, chest pain, Raynaud's phenomenon, and AKI. Diagnosed with a MCTD flare, renal failure, and pneumonia	3/7 intraglomerular thrombi and moderately thickened vessels. Multiple red cell casts within tubular lumina with mild interstitial fibrosis. No evidence of SRC or HUS type TMA	Captopril, MMF, plasma exchange, steroid, HD	Responded to treatment (Cr 1.7 mg/dL)
Vij et al. [22]	Male/21	Oliguria, scleroderma facies, hypertension, and AKI	Bloodless glomeruli, thickening of glomerular capillary walls, interlobular vessels fibrointimal hyperplasia with obliteration of capillary lumen, tubular injury, and interstitial edema	Plasma exchange, HD	HD dependent
Khan et al. [23]	Female/36	Hx of Raynaud's phenomenon, blurry vision, arthralgias, and oliguric renal failure	14 glomeruli were seen which showed nonimmune complex-mediated disease process, ischemic collapse with fibrinoid necrosis. Tubules revealed patchy degeneration with interstitial edema and hyaline casts	Captopril	HD dependent (Cr in range of 2.5–3.0 mg/dl)
Khalil et al. [12]	Male/44	Hypertension, dyspnea, vomiting, Raynaud's phenomenon, skin tightening, and AKI.	2/11 sclerosed glomeruli, remaining glomeruli showing mild to severe capillary collapse. Intimal thickening of blood vessel wall.	HD, captopril	HD dependent (Cr 7.7 mg/dL)
Celikbilek et al. [20]	Female/30	History of sausage-like swellings, Raynaud's phenomenon. Renal dysfunction and pulmonary involvement developed following abortion.	7/12 glomeruli with global sclerosis. Interstitial fibrosis and dense mononuclear cell infiltration. Tubular atrophy. Arterial walls with prominent thickening and hyalinization.	Enalapril, steroids, CTX.	Responded to treatment
Anderson and Vasko [28]	Case 1: female/64 Case 2: male/45	Both cases had features of Raynaud's phenomenon and pulmonary HTN. SRC was provoked by steroids in case 1 and by CHF in case 2.	Case 2: kidney biopsy at autopsy shows renal interlobular arteries and arterioles with edematous, concentric, myxoid intimal proliferation, and thickening almost totally obliterating lumen in a few vessels. These findings were in accordance with SRC.	Enalapril	Response to treatment in both cases (Cr_1_2.03,Cr_2_1.35 mg/dL).
Greenberg and Amato [27]	Female/64	Inflammatory myopathy and bilateral carpal tunnel syndrome who developed AKI following steroid therapy.	Active and severe TMA with extensive mesangiolysis and glomerular capillary wall remodeling with double contours in many glomeruli. Severe arterial and arteriolar sclerosis with fibrin thrombi occlusion.	ACEi, HD	HD dependent (Cr 7.2 mg/dL)
Satoh et al. [6]	Female/47	Raynaud's phenomenon with swollen fingers, sclerodactyly, lymphadenopathy who developed accelerated HTN, AKI and MAHA.	22 glomeruli showed mild ischemic changes. Prominent vascular changes in 2 small arteries, 1/2 with complete occlusion by thrombi and the other with mild intimal proliferation. IF showed faint staining of IgM in the glomerular mesangium.	PSL, PGs, ACEi	Responded to treatment (Cr 1.0 mg/dL)

MCTD, mixed connective tissue disease; SRC, scleroderma renal crisis; AKI, acute kidney injury, Cr, creatinine; HUS, hemolytic-uremic syndrome; TMA, thrombotic microangiopathy; TTP, thrombotic thrombocytopenic purpura; HTN, hypertension; CHF, congestive heart failure; MAHA, microangiopathic hemolytic anemia; IF, immunofluorescence; HD, hemodialysis; MMF, mycophenolate mofetil; ACEi, angiotensin-converting enzyme inhibitor; CTX, cyclophosphamide; PSL, prednisolone; PGs, prostaglandins.

## Data Availability

Data (laboratory and biopsy results) used to support the findings of this case report are included within the article.
